# Gastrotomy for Removal of an Artificial Denture

**Published:** 1885-10

**Authors:** W. D. Miller


### GASTROTOMY FOR REMOVAL OF AN ARTIFICIAL DENTURE.

BY DR. W. D. MILLER.

Whether it is owing to the fact that artificial dentures are more universally worn now than formerly, or that the statistics of teeth-swallowing are more accurately kept, I am unable to say. At all events, the number of accidents from this source, which have been reported in the dental journals, seems, in the last few years, to have undergone a considerable increase.

The greater number of "swallowed" plates have been removed through the oral cavity, without very great difficulty and without any serious results. A few have required Tracheotomy; a considerable number have passed safely through the alimentary tract, while a very few have remained lodged in the stomach.

I am indebted to Dr. N. S. Jenkins, of Dresden, for the report of a case of the last class, where the plate was successfully removed by gastrotomy, this being the second case where this operation has been performed for the removal of swallowed teeth.

"Patient—a barber, twenty-three years old, unmarried, healthy, with no constitutional taint—swallowed a partial upper set (on vulcanite) of six teeth about two weeks ago, a small suction plate with clasp, seemingly some four to five ctm. in diameter. Four days elapsed before he consulted a surgeon. When at last the fact was established that the plate was lodged in the stomach, an operation was decided upon. Before being chloroformed the digestive apparatus was brought into the best possible condition, and a morphine injection given to minimize the tendency to vomit. Incision was made of about ten to fifteen cm., transversely over the pyloric extremity of the stomach. Great care was taken to ligate severed arteries immediately, so that little blood should be lost, and the Lister apparatus and the solution of corrosive sublimate were in constant use. When the stomach was exposed, examination at once showed that the plate was lodged at the pyloric extremity, as had been expected. This portion of the stomach was then drawn out, and ligatures passed through the muscular coat and securely held to prevent the stomach from being drawn back into the abdominal cavity by retching, or any other convulsive movement which might supervene, and then an opening made only just large enough to admit of the extraction of the plate. Great care was also taken not to admit any foreign substance into the stomach. The sewing of the inner coat of the stomach was most beautifully done, the knots of the ligatures being turned inward, that they might find later the easiest exit. Indeed, I could not sufficiently admire the skill with which Dr. Credé tied every ligature, with a perfect appreciation of just how much force was necessary to make a perfect result with every tissue. This is the nicest test of a surgeon's hand. If tied too tight, they tear; if not tight enough, they fail to keep the parts approximate to each other. Exactly an hour was required from the first cut to the final bandaging. At this time of writing (the second day after the operation) the patient is doing as well as could be expected, and there seems to be no reason why a perfect cure should not be looked forward to, and another success be scored by this brilliant young surgeon."

The patient was dismissed perfectly well two weeks later.

